# Perspective: The Place of Pork Meat in Sustainable Healthy Diets

**DOI:** 10.1016/j.advnut.2024.100213

**Published:** 2024-03-18

**Authors:** Adam Drewnowski

**Affiliations:** Center for Public Health Nutrition, University of Washington, Seattle, WA, United States

**Keywords:** fresh pork, protein, national food prices, affordability, greenhouse gas emissions (GHGE), sustainability, protein transition, peak meat consumption, Bennett’s law

## Abstract

The food systems sustainability framework has 4 domains: nutrition, economics, environment, and society. To qualify as sustainable, individual foods and total diets need to be nutrient-rich, affordable, environmentally friendly, and socially acceptable. Pork is the most consumed meat globally, providing high-quality protein and several priority micronutrients. With research attention focused on plant-based diets, it is time to assess the place of pork meat protein in the global sustainability framework. First, not all proteins are equal. The United States Department of Agriculture category of protein foods includes meat, poultry and fish, eggs, beans and legumes, and nuts and seeds. These protein sources have different protein digestibility profiles, different per-calorie prices, and different environmental footprints, measured in terms of greenhouse gas emissions. Second, most analyses of animal-source proteins combine beef, pork, and lamb into a single category of red meat. Beef, pork, and lamb have different nutrient profiles, different protein costs, and different impacts on the environment. Future analyses of nutrient density and monetary and carbon costs of alternative diets would do well to separate pork from beef, lamb, and chicken. There are also different profiles of global food demand. Prior analyses of global Food and Agriculture Organization Statistical Database food balance sheets joined with World Bank country incomes have consistently shown that rising incomes across lower- and middle-income countries (LMIC) create a growing demand for meat to replace traditional plant proteins. Most of the observed increase has been for pork and chicken rather than beef. This ongoing LMIC protein transition toward more animal proteins may be irreversible as long as incomes grow. The present analyses explore the place of pork in sustainable healthy diets worldwide, given the need for high-quality protein and the predictable patterns of global food demand.

**Statements of Significance**

Most studies on sustainable healthy diets do not distinguish among different types of red meat. Separating pork from other red meats, this Perspective explores the place of fresh pork in the sustainability framework, looking at protein content, affordability, and greenhouse gas emissions based on analyses of publicly available data from the United States Department of Agriculture, Food and Agriculture Organization of the United Nations, and the World Bank.

## Introduction

Animal protein from red meat has high monetary and environmental costs [1,2]. The high carbon footprint of meat proteins [2], combined with intensive land and water use, has led to concerns that meat production is not sustainable in the long term [3,4]. With the current global emphasis on plant-forward diets [5,6], the place of animal proteins in human diets needs to be examined more closely with reference to nutrient density, affordability, and impact on the environment [7].

The present Perspective is focused on pork meat. Pork is first in global per capita meat consumption [8]. Pork meat provides high-quality protein, several priority micronutrients, is affordable, and in most societies culturally acceptable [9,10]. Yet pork meat is largely missing from the global nutrition and sustainability discourse and is rarely mentioned in the Dietary Guidelines for Americans [11]. Rather, pork meat in both research and policy documents is typically assigned to the category of red meat [12,13]. As attention turns to the forthcoming United States Dietary Guidelines 2025–2030, it is important to separate pork meat from other protein sources and from other meats, where existing data allow.

Pooling pork, beef, and lamb is very common in nutrition and epidemiology research [[12], [13], [14], [15]]. Food frequency questionnaires often used to support public health policy, typically bundle pork with beef and lamb. “Did you eat beef, pork, or lamb” is a single question on the widely used Fred Hutch food frequency questionnaire [16]. The influential Nurses’ Health Study and other longitudinal cohorts distinguish between fresh and processed meat but do not treat pork meat as a separate category [12,14]. This may have consequences for interpreting data on diets and health outcomes. The failure to distinguish between different types of red meat, corrected only in some recent studies [17], may overlook the unique contribution of pork to the United States diet, with implications for Dietary Guidelines 2025–2030.

The failure to make cost distinctions among different types of red meat may also have food policy consequences. The USDA Thrifty Food Plan (TFP) market basket is the federal estimate of a lowest-cost healthy diet [18]. Its composition is critically important because it is used to allocate food assistance benefits (Supplemental Nutrition Assistance Program), estimated at >$100 billion per year [19]. The last TFP revision (TFP 2021) combined beef and pork into a single category of red meat [18]. Because red meat was more expensive than chicken, the TFP 2021 favored poultry as the main protein source in terms of amounts and allocated expenditures [18]. Calculations based on a lower price for pork led to a different TFP market basket in an independent diet optimization study [20].

The failure to separate greenhouse gas emissions (GHGE) by meat source has implications for the future of food. On the basis of the reported environmental and health impacts of red meat, the influential EAT Lancet report [5] proposed a planetary health diet that was largely plant based, with an estimated 88% of total daily calories coming from cereals, root crops, nuts, legumes, vegetables, and fruit [5]. Pork was limited to 7 g/d (range 0–14 g), the same as beef. Higher amounts were proposed for chicken (29 g) and fish (28 g), both of which were viewed as healthier and more environmentally friendly than red meat.

This Perspective article aims to assess the place of fresh pork in the global sustainability framework, drawing on data from United States sources and from international agencies. The present goal was to examine the sustainability of pork as a source of meat protein, considering nutrition, affordability, environmental impact, and future food demand.

## Databases and Analytical Methods

Analyzing the place of pork in the global diet sustainability framework requires access to data from multiple domains: nutrition and health, economics, and the environment [21,22].

High-quality data on energy and nutrient content of foods came from the USDA databases [23,24]. The dietary component of the nationally representative NHANES 2015–2016 is known as the What We Eat in America (WWEIA) study. The USDA Food and Nutrient Database for Dietary Studies (FNDDS 2015–2016) contains energy and nutrient values for 6581 foods reported as consumed by participants enrolled in the NHANES 2015–2016 study [24]. Listed are individual foods and mixed dishes, prepared in a variety of ways [24]. Food items in the FNDDS 2015–2016 database are aggregated into food groups, food categories, and food subcategories using WWEIA 1-digit, 2-digit, and 4-digit codes [25].

The protein food group in the FNNDS 2015–2016 database includes animal proteins and proteins from plants. The categories are meat, poultry, fish and seafood, eggs, as well as beans, peas and legumes, soy products, and nuts and seeds [26]. Dairy is another source of dietary protein, with categories defined as milk, yogurt, and cheese. WWEIA codes also include the grains group and the large category of mixed dishes, such as sandwiches, soups, and mixed foods that could be meat or plant based [25]. Not included in analyses were alcoholic beverages, vegetables and fruit, condiments, and snacks and sweets, all of which contained little protein.

Mean national retail prices for 3231 FNDDS food codes were based on the 2015–2016 Purchase to Plate Price Tool [26] and came from the TFP 2021 Supplemental files [18]. As reported in the TFP 2021 [18], the more costly foods and foods purchased by NHANES participants with incomes at >350% of federal poverty had been excluded. To calculate the TFP 2021, the USDA adjusted the 2015–2016 prices for inflation to June 2021 [18].

Previously used data on GHGE for FNDDS 2015–2016 database foods [27] came from the Food Impacts on the Environment for Linking to Diets (dataFIELD) database and from a systematic review of life cycle assessments (LCA) published between 2005 and 2016 [28,29]. The GHGE estimates were averaged across studies and were matched to commodities in the 2010 United States Environmental Protection Agency (US EPA) Food Commodity Intake Database (FCID) [28,29]. The FCID provides information on the amount of >500 food components in each food reported as consumed by participants enrolled in NHANES [29]. GHGE data for FNDDS 2015–2016 database foods were merged with energy, nutrients, and national food prices data using food identification codes.

Data to illustrate the place of pork meat in the global food supply came from the FAO of the United Nations FAOSTAT data repositories [30,31]. At the global level, FAOSTAT [31] provides food balance sheets for selected commodities (bovine meat and pigmeat). Those data are limited to items that are part of formal trade and enter the retail market; informal commerce is not included. Food balance sheets are also used to calculate amounts of total protein, animal protein, and plant protein (in kg/capita/y) that are available for human consumption. Despite their many limitations, FAO food balance sheets and other food supply data are routinely used as proxies for human food consumption [5,32].

Data on country incomes came from the World Bank [33]. The World Bank classifies economies for analytical purposes into 4 income groups: low, lower-middle, upper-middle, and high income. The data are expressed as gross national income per capita in United States dollars, converted from local currency using the World Bank Atlas method to smooth exchange rate fluctuations [33]. The present analyses were based on 164 countries that had both FAO data and World Bank incomes for the year 2019. Both sets of data are publicly available and can be downloaded from the FAO and World Bank websites, respectively.

## **The Place of Pork in the Sustainability Framework**

A review of the literature, combined with some original analyses, has led to these perspectives.

### Pork meat is an excellent protein source

First, the red meat category was separated into beef, pork, lamb, and cured meats. Table 1 shows protein content in g/100 g, energy density in kcal/100 g, and the amount of dietary energy needed to obtain 50 g of protein [100% reference daily value (DV)] by food category. This type of nutrients to energy calculation has been used before [34]. Analyses of energy and protein content used 1-way analysis of variance (ANOVA) with Bonferroni correction for planned post hoc tests. The mean energy density of all meats was ∼200–300 kcal/100 g. The mean amount of protein in pork items was 27.6 g/100 g, not significantly different from red meat or poultry but significantly above the other food categories tested.

**TABLE 1:** Protein content (g/100 g), energy density (kcal/100 g), and estimated kcal needed to obtain 50 g of protein by food group and category in the Food and Nutrient Database for Dietary Studies

Food category	*N*	Protein content (g/100 g)		Energy density (kcal/100 g)		Kcal for 50 g (100% DV) of protein	
		Mean	SEM	Mean	SEM	Mean	SEM
Beef	81	27.27	0.45	228.75	6.04	441.47	20.96
Pork	83	25.39	0.64	247.66	8.81	535.16	50.05
Lamb	23	25.22	0.49	245.30	14.53	496.28	35.18
Cured meats	46	23.96	1.18	284.85	21.29	869.96	293.79
Poultry	224	23.18	0.28	214.38	3.50	496.45	14.32
Seafood	434	21.01	0.35	177.13	2.73	447.96	8.51
Animal protein	109	19.53	0.7	229.88	8.39	693.84	38.10
Plant protein	174	13.71	0.58	361.96	15.92	2365.15	900.69
Eggs	151	11.84	0.17	169.20	4.52	712.13	15.15
Mixed dishes	1985	8.92	0.11	172.18	1.87	1152.25	14.87
Milk and dairy	239	8.76	0.57	135.19	7.67	1181.44	94.19
Grains	624	6.84	0.14	253.81	4.35	2250.26	169.01

Abbreviation: DV, daily value..

Beef, pork, lamb, poultry, and seafood were high in protein per 100 g and delivered 100% DV of protein for the least calories. At the other extreme, getting 50 g of protein from grains would require (in theory) >2000 kcal/d. The protein leverage hypothesis [35] suggests that humans eat to satisfy protein needs, and that foods will be consumed until protein needs have been met, regardless of energy content. If the protein leverage hypothesis is correct, that could potentially lead to an over-consumption of some foods (for example, starches and grains) when their protein content is low.

**FIGURE 1:**
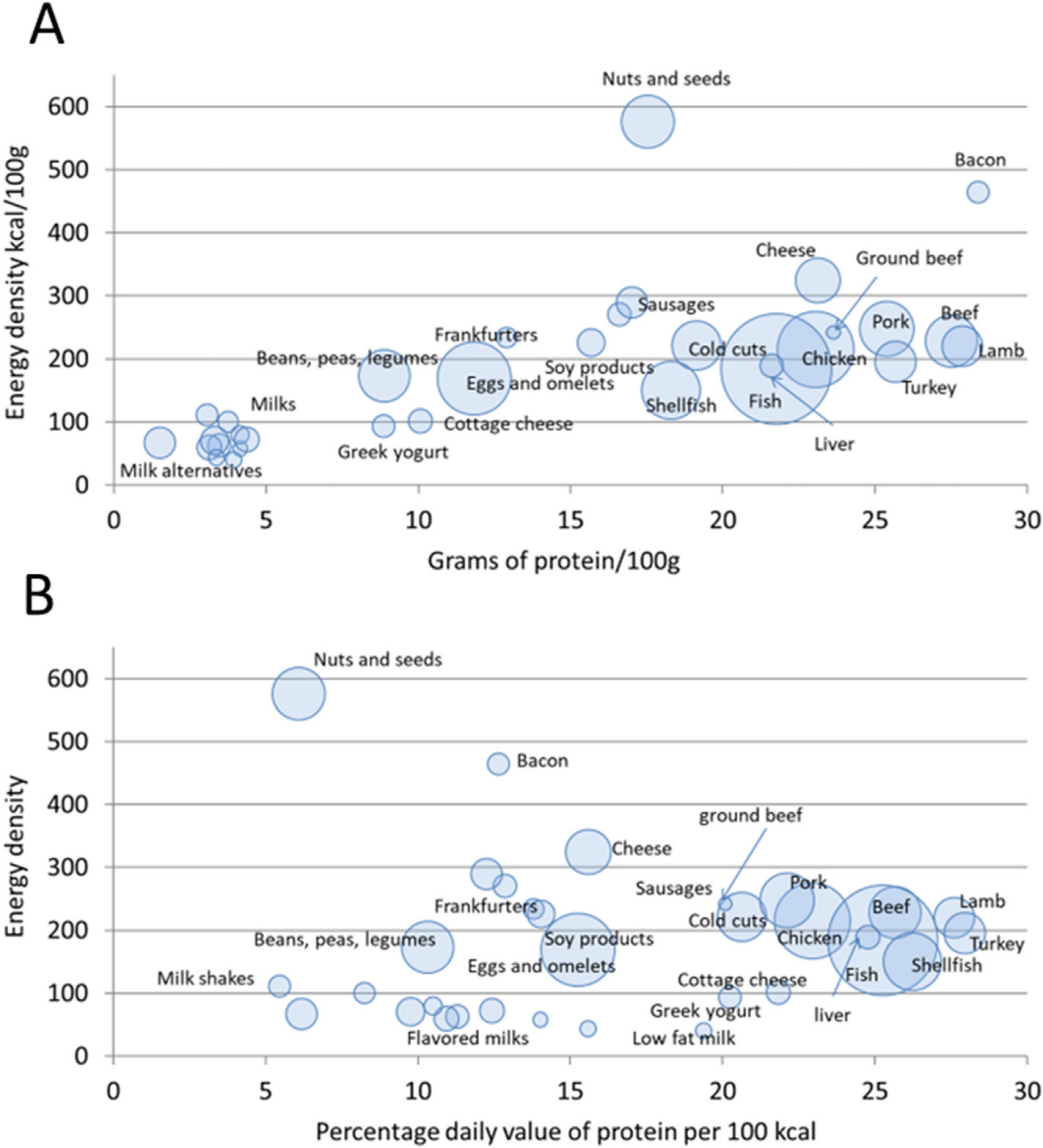
A scatterplot of mean grams of protein/100 g (A) and percent daily value (% DV) for protein (B) plotted against mean energy density (kcal/100 g) for protein foods, milk, and dairy in the FNDDS 2015–2016 database. Size of the bubble corresponds to the number of food items in each category. DV, daily value; FNDDS, Food and Nutrient Database for Dietary Studies.

Subsequent analyses used finer WWEIA subcategories, separating beef and ground beef, chicken and turkey, fish and shellfish, and milk, yogurt, and cheese. Plant proteins were separated into beans, peas and legumes, soy products, and nuts and seeds. Protein content was also calculated for bacon, cold cuts, sausages, and frankfurters. Figure 1A shows the relation between mean grams of protein per 100 g plotted against mean energy density by WWEIA subcategory.

Most animal-source food categories in the FNDDS 2015–2016 database had a mean of >20 g of protein per 100 g. Foods that were highest in protein were pork, turkey, beef, and lamb dishes. Fresh pork items contained a mean of 25.4 g of protein per 100 g, as compared with 27.3 g/100 g for beef and 23.2 g/100 g for chicken. For comparison, the protein content of Greek yogurt was ∼10 g/100 g, whereas cheeses contained ∼20 g/100 g protein. Foods that provide >20% DV per serving for a given nutrient are deemed to be excellent sources of that nutrient.

The amount of dietary energy needed to supply 50 g of protein depends on energy density (kcal/100 g). Meats and meat dishes contained between 150 and 300 kcal/100 g; bacon contained closer to 500 kcal/100 g, whereas nuts and seeds contained close to 600 kcal/100 g. The protein content of plant foods was generally below 10 g/100 g (except for processed soy) whereas the energy and carbohydrate content were high. As a result, the protein-to-calories ratio for nuts and seeds was now below that for beans and legumes, as shown in Figure 1B. Foods with maximum protein percent DVs (%DV) per 100 kcal of food were pork, turkey, beef, and lamb. Mixed dishes, nuts, and seeds (and bacon) provided less protein per calorie because of their high energy density.

The present data were not corrected for Protein Digestibility Corrected Amino Acid Score (PDCAAS) [36]. In general, based on available (but still limited) PDCAAS data, meat, milk, and eggs have more digestible protein per 100 g than plant-based proteins, including grains, seeds, and nuts. The USDA school lunch program requires plant proteins to have PDCAAS of >80% [37]. Pork, beef, chicken, seafood, eggs, and dairy have favorable PDCAAS and Digestible Indispensable Amino Acid Score (DIAAS) values. DIAAS data for FNDDS foods are not yet available.

### Pork meat is an affordable protein source

**FIGURE 2:**
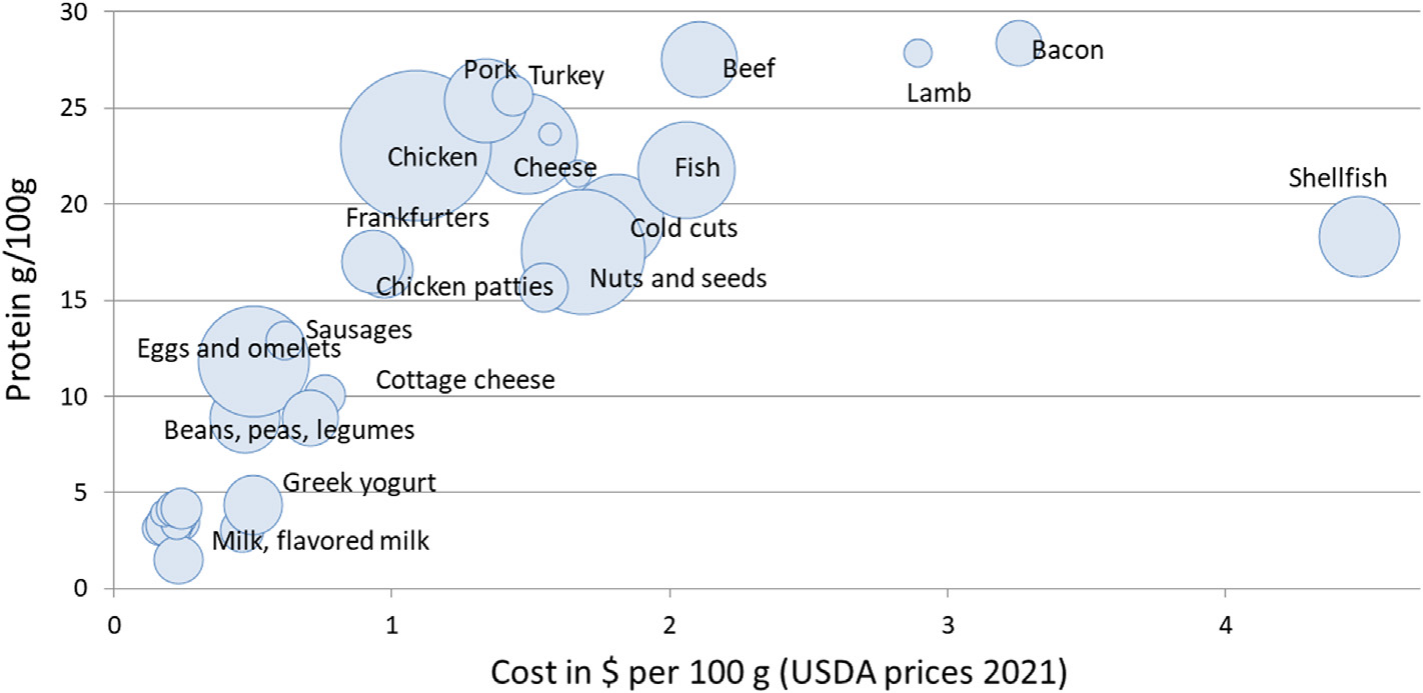
Scatterplot of mean price per 100 g ($/100 g) plotted against mean energy density (kcal/100 g) of protein foods, milk, and dairy in the FNDDS 2015–2016 database. Size of the bubble corresponds to the number of food items in each category. FNDDS, Food and Nutrient Database for Dietary Studies.

Food prices that were obtained from the TFP 2021 Supplemental data files [18] were limited to ∼3000 foods in all. Figure 2 shows protein content in g/100 g plotted against mean prices per 100 g for each protein food category and subcategory. There was a clear separation in food prices between shellfish and other proteins and then between pork, beef, and lamb. Mean national prices for pork meat were below beef and fish and very close to chicken and turkey.

**FIGURE 3:**
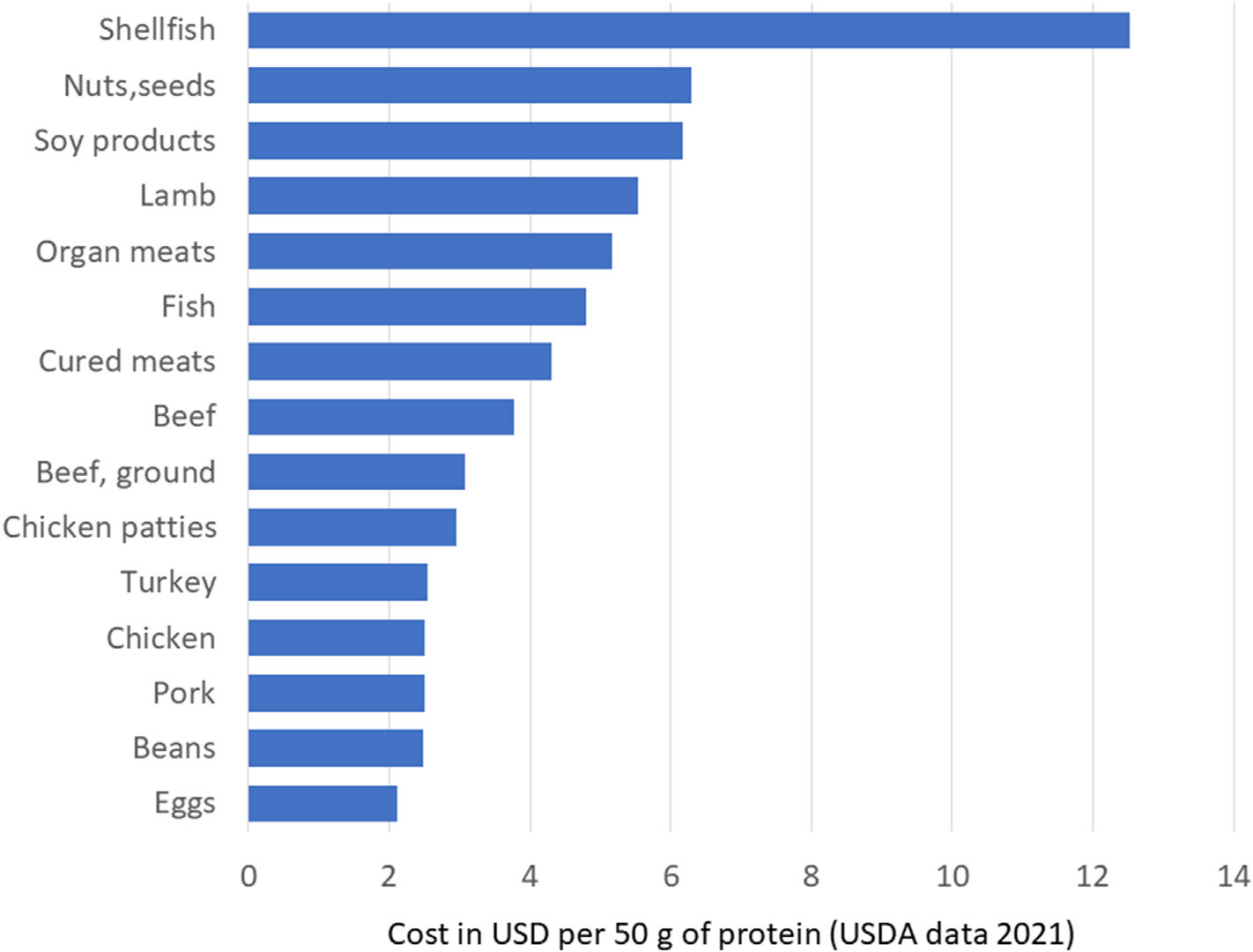
Monetary cost in United States dollars per 50 g of protein (100% DV) shown in decreasing order. National prices are from the USDA 2021 Thrifty Food plan. DV, daily value.

Figure 3 shows the cost of protein foods in the TFP 2021 database, expressed per 50 g of protein (that is 100% DV). These are the same prices that had been used in the construction of the revised TFP 2021. The protein foods, animal and plant, were now separated into categories and subcategories based on USDA codes. Shellfish were the most expensive protein source, as had also been noted in the TFP 2021 report [18]. Once the red meat category was separated into components, fresh pork was closer in price to chicken and beans than it was to lamb or beef. The price for 50 g of protein from pork was not significantly different from that of poultry and eggs but significantly below other types of meat.

This price differential has consequences for the design of affordable healthy food plans. The TFP 2021 [18] market basket, developed by the USDA Center for Nutrition Policy and Promotion (CNPP), is the current federal estimate of the lowest-cost healthy and nutrient-adequate food plan, suitable for a standard family of 4. The CNPP used the cut-point of <4.5 g/100 g of saturated fat (and zero added sugar) to identify higher nutrient density meats and then searched for lower-cost items within each category. Where meat prices varied, further categories were created based on lower or higher national food prices. This was to allow the TFP optimization model to select a market basket of lowest-cost protein foods for a nutritious diet.

Identified by the USDA CNPP as higher nutrient density and lower-cost meats were numerous cuts of fresh pork: pork chop (baked, broiled, stewed, and fried), pork roast, and pork steak/cutlet. Beef liver was listed as well. Lower nutrient density meats (with saturated fat >4.5 g/100 g) listed in Supplemental data files were pork roast, pork spareribs with barbecue sauce, ground pork, pork steak/cutlet (baked and broiled), and pork chop (breaded and baked, broiled, and fried). Also on the list were beef steak (breaded and baked, fried) and beef pot roast.

Similar criteria were used to identify higher nutrient density poultry. Higher nutrient density dairy had no >1% fat and zero added sugar. All seafood had high nutrient density.

The higher nutrient density and lower protein foods (*n* = 596) in the TFP 2021 optimization model are shown in Table 2. Milk and dairy products, mixed dishes, and grain foods are also shown for comparison. As previously observed, the protein content of beef, pork, and poultry was significantly above the other WWEIA categories. The saturated fat content of pork, beef, and poultry was significantly lower than for plant proteins (nuts) and egg dishes in the database.

**TABLE 2:** Nutrient content of foods identified as higher nutrient density in the Thrifty Food Plan 2021 (*N* = 596)

Food category	*N*	Protein (g/100 g)	SEM	Saturated fat (g/100 g)	SEM	Sodium	SEM	Potassium	SEM
Beef	15	28.72∗	0.54	2.65∗	0.19	382∗	8	328∗	15
Pork	16	27.58∗	0.37	2.41∗	0.22	511∗	16	437∗	16
Poultry	90	23.62∗	0.45	2.36∗	0.11	425∗	11	269	5
Plant proteins	54	17.04	0.92	6.67	0.59	167	26	605	26
Eggs	17	11.47	0.30	4.53	0.30	307	16	151	6
Mixed dishes	294	7.65	0.24	2.22	0.12	305	7	179	4
Milk and dairy	31	4.73	0.43	1.28	0.21	64	6	183	10
Grains	75	7.73	0.45	0.92	0.12	349	25	233	17

∗ Denotes those categories that are not significant from each other but different from the rest.

Subsequent modeling analyses conducted by MS-Nutrition [20] replicated CNPP procedures but separated pork from beef to create 5 low-cost healthy food plans. Once pork was an independent modeling category, the TFP algorithm preferentially selected fresh pork. Model 1 replicated the TFP exactly. Models 2 and 3 showed that diets with fresh pork as the only source of meat protein were both healthy and low cost. Model 4 showed that pork could substitute for chicken with no change in nutrient quality and no increase in market basket cost.

Calculations based on pork as a separate category can help determine the cost of affordable nutrition in the United States [38,39]. Modeling analyses that replicated the TFP 2021 firmly established pork as a component of practical, healthy, and budget-conscious diets that were consistent with United States eating habits. Pork (along with chicken) provided the lowest-cost way to reach 100% DV for dietary protein. The versatility of pork meat contributes to its potential as a protein staple in many regional dietary patterns that include vegetables, fruits, whole grains, oils, nuts, and seeds.

### Pork meat has low greenhouse gas emissions

Animal proteins have a greater carbon footprint as compared to plant proteins, with most of the calculations pointing to red meat [1,2]. However, not all data used to support public policy were context specific or have clearly distinguished among different types of red meat. For example, the very influential article by Poore and Nemecek [40] was based on a composite of international numbers for 127 countries that did not necessarily reflect the best practices of livestock management in the United States. On the other hand, Poore and Nemecek [40] did distinguish between ruminants and nonruminants and made further useful distinctions between the relative environmental impacts of meat from beef herd, cattle herd, poultry, and pork.

**FIGURE 4:**
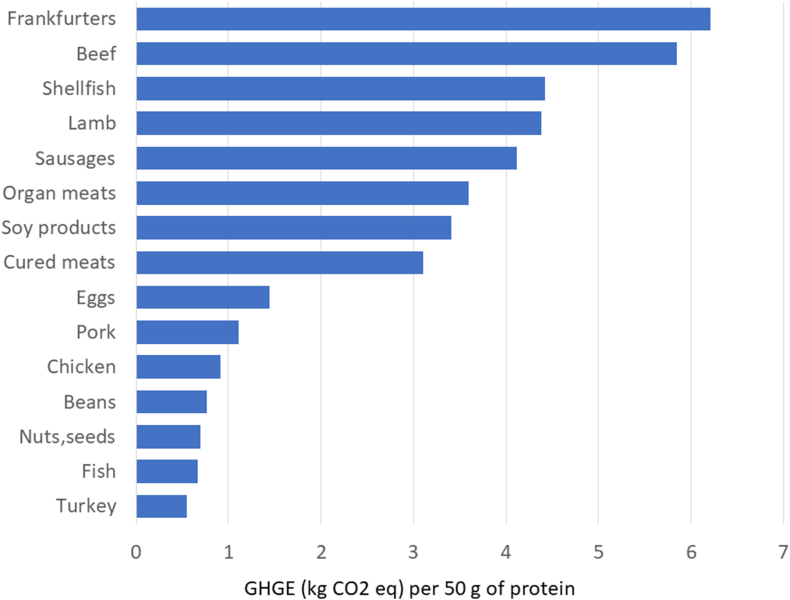
Estimated mean greenhouse gas emissions (GHGE) in CO_2_ eq kg per 50 g of protein (100% DV) plotted against mean protein content of foods in g/100 g by category. CO_2_ eq, carbon dioxide equivalents.

The present analyses used GHGE estimates from previous studies [27,28] on carbon footprint of popular United States diets. Protein foods were separated into categories and subcategories, and the data were expressed per 50 g of protein. Figure 4 shows mean GHGE estimates in kg CO_2_ per 50 g of protein. Fresh pork was significantly below beef and lamb, the other red meats, and was closer to eggs, chicken, and beans. One-way ANOVA with post hoc Duncan’s test placed pork in the lowest category of GHGE values. It should be noted that the estimated GHGE values for protein from grains and nuts will most likely increase, once PDCAAS values are considered.

Treating pork as a separate category may reshape our ideas about the environmental cost of animal protein, typically measured in terms of GHGE per product weight or volume (kg or L). The present analyses followed the same convention in expressing GHGE per kg of food. A recent FAO report [41] explored the use of alternative nutrition-relevant functional units that might better serve nutrition-relevant LCA. One proposal was to base calculations on the amount of protein (DV = 50 g/d) or on a composite nutrient density score. The carbon footprint of pork could be assessed using 100 g of protein as a functional unit [38]. Alternatively, the calculations can be based on protein quality [42], or a nutrient density score as proposed by the FAO [41]. However, this is a rapidly advancing field, and any calculations ought to be viewed as preliminary and nondefinitive.

## The Place of Pork in the LMIC Nutrition Transition

Changes in population diet structure that occur during economic development are commonly referred to as the nutrition transition [43]. Although the nutrition transition may be primarily income driven, other factors such as urbanization, demographics, and employment also play a role [43,44]. Bennett’s law [45] is the name given to the observation that richer countries and more affluent consumers abandon root crops and cereals to seek out more varied and more nutrient-dense diets with more vegetables, fruit, and dairy, but especially meat. The proportion of energy from starchy staples, cereals, and potatoes, declines, whereas the proportion of energy from meats increases [45]. Effectively, Bennett’s law predicts that plant-based proteins will be replaced by animal proteins as an inevitable consequence of economic growth.

Laws of economics can serve to predict future global food demand [46,47]. In general, richer countries and more affluent consumers will seek out calories that are more expensive and more nutrient rich.

**FIGURE 5:**
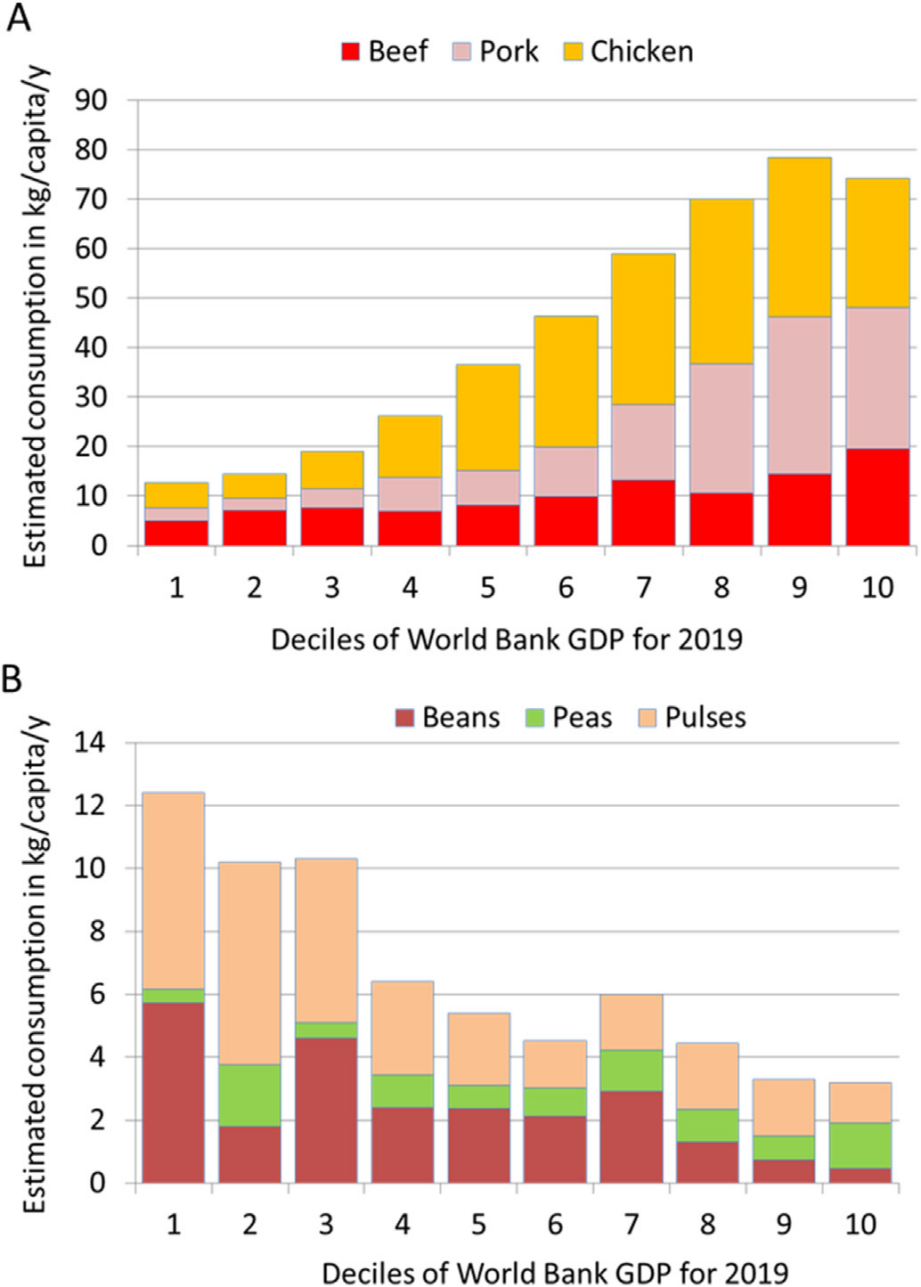
The relation between beef, pork, and chicken (A) and pulses, peas, and beans (B) available for human consumption in 2019 by deciles of GDP per capita by country. Food balance sheets from FAO of the United Nations. GDP data from the World Bank. GDP, gross domestic product.

This can be documented by merging FAOSTAT data for energy from plant and animal proteins in calories/capita/day with Work Bank incomes for 2019 for the same countries. The present analyses were conducted by gross domestic product deciles and not by World Bank income groups. Figure 5A provides yet another confirmation of Bennett’s law showing that the availability of beans, peas, and pulses for human consumption in the 2019 FAO data declines rapidly at higher incomes. Figure 5B shows a corresponding increase in the availability of pork, chicken, and beef calculated in kg/capita/year. The rapid increase in meat consumption occurring among middle-income countries has also been observed in other studies. There has been an explosive growth in poultry (chicken) consumption, followed by pork [48]. By contrast, beef has shown less growth [48,49]. Although future meat demand can be difficult to predict [50], current OECD models [49] project a 95% increase for animal protein, compared with only 18% for starchy crops. Although rising meat consumption across the lower- and middle-income countries (LMIC) is clearly linked to World Bank incomes, other factors can also influence diet structure, including tradition, religion, and culture [51], food prices, and concerns with health and the environment [52].

Recent efforts to promote plant-based and planetary health diets across the LMIC [4,5] have raised concerns because they may conflict with the laws of economics and ignore local and territorial preferences and food cultures. For high-income countries that may have reached peak meat consumption [53], international agencies and local governments aim to reduce meat consumption to improve diet quality and population health [54]. Conversely, agencies and local governments in lower-income countries aim to increase meat and dairy consumption, also to improve diet quality and population health [55]. The present reanalyses of global FAO and World Bank data confirmed that the growing LMIC demand for animal protein was directed mostly toward chicken and pork, rather than beef. It is something of a public health paradox that higher-income countries aim to replace pork with beans, whereas lower-income countries are replacing beans with pork.

It is worth noting that the EAT Lancet proposal to limit meat consumption [5] runs counter to the economic trends and the protein transition that is observed in LMIC [31,33]. The growing LMIC demand for animal-sourced foods, mainly meat, is 1 way to address multiple protein and other nutrient needs [56,57]. The traditional and largely plant-based LMIC diets are still associated with multiple micronutrient deficiencies. Studies have identified LMIC priority micronutrients as high-quality protein, iron, zinc, calcium, vitamin A, B vitamins, and vitamin D [58]. Recent analyses have pointed to shortfalls in the EAT Lancet planetary health diet for vitamin B12, calcium, iron, and zinc [59]. LMIC meat demand is projected to increase substantially over the next decade.

The global dietary shift from plant to animal proteins, previously characterized as a protein transition [44,45], can be viewed as a subset of the broader nutrition transition [43]. Although largely income-driven, the protein transition has additional social and cultural components [51]. The choice of specific animal proteins (beef, pork, chicken, or dairy) can vary widely depending on geographic region, tradition, religion, or culture. Not all cultures or all religions consume pork.

In conclusion, livestock systems have long been associated with higher land, water, and energy use and there are fears that they may not be sustainable for much longer [1,2]. Health and environmental concerns related to meat production are among the main reasons behind the current initiatives to promote plant proteins on a global scale [5]. However, the growth in animal proteins has been in chicken and pork, not beef. We may need to recalibrate our comparisons and pay attention to local contexts and to specific food groups. The present analyses, separating pork from other meats, can help refine the definition of sustainable healthy diets [60].

Pork meat is a source of affordable high-quality protein and may have a lower environmental (GHGE) impact than previously supposed. Separating pork from other red meat may reshape our ideas about diets and health and the environmental cost of meat production. The present analyses of USDA nutrient composition and national food prices data treated pork as a separate category. Whereas the amounts of protein in pork, beef, lamb, seafood, and chicken were comparable, pork and chicken had a clear price advantage. Achieving affordable nutrient density has been identified by national and international agencies as a priority area [61,62].

The rising LMIC demand for meat protein foods may be hard to stop and harder to reverse, given that it is consistent with Bennett’s law. Only a handful of the richest countries have achieved what has been called peak meat consumption [53]. The present analyses of FAO data, consistent with many other studies, confirmed that higher country incomes were associated with diets with less starchy staples and more meat, notably chicken and pork.

## Author contributions

The author’s contributions were as follows – AD: was solely responsible for design, writing, and final content and has read and approved the manuscript and has read and approved the final version.

## Conflict of interest

AD is the original developer of the Naturally Nutrient Rich (NNR) and the Nutrient Rich Food (NRF) nutrient profiling models and a member of scientific advisory panels for The National Pork Board, Nestlé, FrieslandCampina, BEL, and Carbohydrate Quality Panel supported by Potatoes USA and has worked with Ajinomoto, FoodMinds, KraftHeinz, Nutrition Impact LLC, Nutrition Institute, PepsiCo, and Samsung on quantitative ways to assess nutrient density of foods.

## Funding

Analyses of publicly available USDA, FAO, and World Bank data were supported by the National Pork Board. The funders were not involved in the development of databases, analytical models, data analysis or interpretation, manuscript preparation or the decision to submit the manuscript for publication.
